# The association between early-onset cardiac events caused by neoadjuvant or adjuvant chemotherapy in triple-negative breast cancer patients and some novel autophagy-related polymorphisms in their genomic DNA: a real-world study

**DOI:** 10.1186/s40880-018-0343-7

**Published:** 2018-12-04

**Authors:** Binliang Liu, Tao An, Meiying Li, Zongbi Yi, Chunxiao Li, Xiaoying Sun, Xiuwen Guan, Lixi Li, Yanfeng Wang, Yuhui Zhang, Binghe Xu, Fei Ma, Yixin Zeng

**Affiliations:** 1 Department of Medical Oncology, National Cancer Center/National Clinical Research Center for Cancer/Cancer Hospital, Chinese Academy of Medical Sciences and Peking Union Medical College, Beijing, 100021 P. R. China; 2 State Key Laboratory of Cardiovascular Disease, Heart Failure Center, Fuwai Hospital, National Center for Cardiovascular Disease, Chinese Academy of Medical Sciences and Peking Union Medical College, Beijing, 100037 P. R. China; 3 Shandong Cancer Hospital and Institute, Shandong University, Jinan, 250117 Shandong P. R. China; 4 State Key Laboratory of Molecular Oncology, Cancer Institute/Hospital, Chinese Academy of Medical Sciences and Peking Union Medical College, Beijing, 100021 P. R. China; 5 Department of Medical Oncology, Cancer Hospital of Huanxing, Beijing, 100065 P. R. China; 6 Department of Comprehensive Oncology, National Cancer Center/Cancer Hospital, Chinese Academy of Medical Sciences and Peking Union Medical College, Beijing, 100021 P. R. China; 7 Chinese Academy of Medical Sciences and Peking Union Medical College, Beijing, 100730 P. R. China; 8 Collaborative Innovation Center for Cancer Medicine, State Key Laboratory of Oncology in South China, Sun Yat-sen University Cancer Center, 651 Dongfeng Road East, Guangzhou, 510060 Guangdong P. R. China

**Keywords:** Triple-negative breast cancer, Chemotherapy, Cardiotoxicity, Autophagy, Single nucleotide polymorphisms

## Abstract

**Background:**

An increasing number of cancer patients die of cardiovascular diseases. The cardiotoxicity of chemotherapy is particularly important in triple-negative breast cancer (TNBC) with limited therapeutic options. Cardiac autophagy is an important mechanism of cardiotoxicity. This research was aimed to investigate the cardiotoxicity of chemotherapy in TNBC, screen the susceptible population, and determine the relationship between cardiotoxicity and autophagy-related polymorphisms.

**Methods:**

From a total of 2450 stage I-III TNBC patients, 147 met the inclusion criteria and finally recruited. Electrocardiography (ECG) was performed before most chemotherapy cycles, and echocardiography (UCG) was performed according to clinical needs. All ECG and UCG records were re-interpreted by cardiologists at the National Center for Cardiovascular Disease, Fuwai Hospital. According to the National Center for Biotechnology Information and the Catalog of Somatic Mutations in Cancer database, we selected 25 single nucleotide polymorphisms (SNPs) related to autophagy and genotyped the 147 TNBC patients. Paired-sample *T* tests, Chi squared tests, and logistic regression models were employed for the analysis.

**Results:**

Only 46 (31.3%) patients had normal ECG records after every chemotherapy cycle. Among the 16 patients who underwent UCG, 2 (12.5%) had a reversible decrease of left ventricular ejection fraction. The use of anthracyclines and excessive alcohol consumption were risk factors of ECG abnormalities. With the continuation of chemotherapy, heart rate gradually increased. Anthracyclines were associated with QRS duration abnormalities (*P *= 0.043). After genotyping for 25 autophagy-related SNPs, we found that the G allele of autophagy-related 13 (*ATG13*) rs10838611 was significantly associated with ECG abnormalities (odds ratio = 2.258, 95% confidence interval = 1.318–3.869; *P *= 0.003).

**Conclusion:**

ECG abnormalities caused by chemotherapy are common in the real world. Autophagy-related SNPs are associated with chemotherapy-induced cardiotoxicity, thereby providing new evidence for autophagy as a cause of chemotherapy-induced cardiac damage.

**Electronic supplementary material:**

The online version of this article (10.1186/s40880-018-0343-7) contains supplementary material, which is available to authorized users.

## Background

With the application of many new antineoplastic drugs and improved treatment options, the number of cancer survivors is rapidly rising. However, with increasing life expectancy, cancer patients also receive more cycles or more types of treatment, resulting in an increase in the cumulative dose of some effective drugs that are implicated in long-term adverse cardiovascular outcomes [1]. Triple-negative breast cancer (TNBC) accounts for 15% of breast cancers and exhibits special biological and clinicopathological characteristics, as well as high proliferation, poor differentiation, and poor prognosis [2]. In the absence of approved targeted therapy, chemotherapy containing anthracyclines and taxanes remains the mainstay of the limited treatment options for TNBC at present [2, 3].

Anthracyclines have high efficacy on solid tumors and hematological malignancies. However, many studies have shown cardiotoxicity of anthracyclines and some molecular targeted drugs [4–6]. With repeated cycles of chemotherapy, cumulative doses gradually increase in TNBC patients, and so does the risk of cardiac adverse events [7, 8]. At present, the widely accepted consensus is that although cardiac damage may appear as early as the first dose, the risk of adverse events increases with increasing cumulative doses; when the cumulative dose of doxorubicin (ADM) reaches 250 mg/m^2^ or that of epirubicin (EPI) reaches 600 mg/m^2^, the risk of adverse events is significantly elevated [7–9]. In addition to anthracyclines, trastuzumab, tyrosine kinase inhibitors (e.g., lapatinib), antimetabolite drugs (e.g., 5-fluorouracil [5-FU], capecitabine), cyclophosphamides, and antimicrotubule agents (e.g., docetaxel, paclitaxel) can cause mild or severe cardiac damage via different pathophysiological mechanisms [7, 8].

Many mechanistic studies have been conducted, and hypotheses have been proposed about the cardiotoxicity of antineoplastic drugs; free iron-based or radical-induced oxidative stress [4] and dysregulation of autophagy [10] have been investigated. Autophagy has dual functions and plays important roles in homeostasis, and thus the regulation of autophagy is very important. In the heart, autophagy may play a role in adaptation to hemodynamic stress and the prevention of age-dependent dysfunction, but it can also cause cardiac hypertrophy [11]. Autophagy induced by drugs, especially chemotherapy agents, is a new area of study. Most studies concluded that anthracyclines up-regulate cardiac autophagy [10], whereas trastuzumab down-regulates autophagy [12]. Cardiac adverse events, especially type I cardiomyopathy caused by some antineoplastic drugs, is often progressive and irreversible [13]; therefore, early detection and prevention are of great importance. Early-onset cardiac damage may result in arrhythmia, a reduction in left ventricular ejection fraction (LVEF; a significant decrease corresponds to > 10% drop) and electrocardiographic changes [7–9]. Therefore, because of the advantages of the techniques, it is necessary to perform electrocardiography (ECG) and echocardiography (UCG) regularly to monitor acute cardiotoxicity; however, there are still many mysteries about the relationship between electrocardiographic or echocardiographic changes and cardiotoxicity, as well as their associations with prognosis [7, 8].

Close attention must be paid to cardiotoxicity of chemotherapy. In addition to performing early diagnosis, we are interested to establish a prognostic model of cardiac events at the molecular level. Although several single nucleotide polymorphisms (SNPs) have been used to evaluate the associations between genetics and cardiac events in cancer patients, relevant studies have usually been performed in patients on anthracyclines [14]. Based on previous studies and databases, we selected 25 candidate SNPs to determine their relationships with abnormalities on ECG or UCG records during chemotherapy. The purpose of this study was to investigate cardiac events caused by neoadjuvant or adjuvant chemotherapy in TNBC patients and to determine the association between abnormalities on ECG or UCG records and certain autophagy-related SNPs.

## Materials and methods

### Study subjects

We reviewed the electronic records of breast cancer patients treated at the Cancer Hospital, Chinese Academy of Medical Sciences (Beijing, China) between January 2008 and December 2015. The patient selection criteria were as follows: (1) volunteered to participate in this study; (2) female patients; (3) pathologically diagnosed with stage I–III TNBC; (4) complete clinicopathological data including age, tumor size, TNM stage, pathological type, adjuvant chemotherapy, past medical history, family history, smoking and drinking history; (5) available reports of ECG and/or UCG; and (6) sufficient blood samples for SNP genotyping. Exclusion criteria were as follows: (1) symptomatic heart failure; (2) acute phase of coronary heart disease; and (3) previous history of fatal arrhythmia. We followed all patients until August 6, 2017. This study was approved by the Institutional Review Boards of the Cancer Hospital, Chinese Academy of Medical Sciences (No. CH-BC-019).

### Breast cancer subtype definition

Estrogen and progesterone receptor statuses were evaluated based on immunohistochemistry (IHC) of formalin-fixed, paraffin-embedded breast cancer tissue samples obtained from the patients. IHC was performed with anti-estrogen receptor and anti-progesterone receptor antibodies. A positive estrogen or progesterone receptor status was defined by nuclear staining of more than 1% cells based on the guidelines of American Society of Clinical Oncology (ASCO) and College of American Pathologists (CAP) released in 2010. To determine the human epidermal growth factor 2 (HER2) status, IHC was performed or gene amplification was determined using fluorescence in situ hybridization (FISH). Tumors are classified as HER2-positive if scored as 3+ for HER2 in ≥ 10% tumor cells as demonstrated by IHC or if gene amplification is demonstrated by FISH. Tumors negative for estrogen and progesterone receptors and HER2 were defined as TNBCs.

### Exposure dose of chemotherapeutic drugs

All TNBC patients received adjuvant/neoadjuvant treatment. The chemotherapy regimens were as follows: (1) the EC regimen [EPI 90 mg/m^2^ or pirarubicin (THP) 40–50 mg/m^2^ on day 1 and cyclophosphamide (CTX) 600 mg/m^2^ on day 1, repeated every 21 days for 4–6 cycles]; (2) the EC-T regimen [EPI 90 mg/m^2^ and CTX 600 mg/m^2^ on day 1, repeated every 14 or 21 days for 4 cycles, followed by docetaxel (DTX) 75 mg/m^2^ on day 1, repeated every 21 days for 4 cycles, or paclitaxel (PTX) 175 mg/m^2^ on day 1, repeated every 14 or 21 days for 4 cycles]; (3) the ET regimen (EPI 75 mg/m^2^ or THP 40–50 mg/m^2^ on day 1 and DTX 75 mg/m^2^ or PTX 175 mg/m^2^ on day 2, repeated every 21 days for 6 cycles); (4) the TAC regimen (EPI 75 mg/m^2^ or THP 40–50 mg/m^2^, CTX 500 mg/m^2^, and PTX 175 mg/m^2^ or DTX 75 mg/m^2^ on day 1, repeated every 21 days for 6 cycles); (5) the CAF regimen [CTX 500 mg/m^2^ on day 1, EPI 75 mg/m^2^ or THP 40–50 mg/m^2^ or ADM 50 mg/m^2^ on day 1, 5-fluorouracil (5-FU) 500 mg/m^2^ on days 1 and 8, repeated every 21 days for 6 cycles]; (6) the TC regimen (DTX 75 mg/m^2^ or PTX 175 mg/m^2^ and CTX 600 mg/m^2^ on day 1, repeated every 21 days for 4 cycles); (7) the carboplatin-taxane regimen [DTX 75 mg/m^2^ or PTX 175 mg/m^2^ on day 1, and carboplatin (CAPE) area under receiver-operating curve (AUC) = 5 mg/mL on day 2, repeated every 21 days for 6 cycles].

The EC and CAF regimens were classified as anthracycline-based regimens; the TC regimen was a taxane-based regimen; the EC-T, ET, and TAC regimens were classified as anthracycline–taxane-based regimens.

### Electrocardiography and echocardiography

Standard 12-lead ECG was performed before chemotherapy and after most chemotherapy cycles. As we mainly study early-onset cardiac events, we chose to analyze ECG records after chemotherapy. Abnormal electrocardiogram refers to the presence of new abnormalities after any chemotherapy cycle compared with baseline electrocardiogram, regardless of whether the abnormality disappeared during the subsequent chemotherapy cycle. For patients with abnormal electrocardiograms, UCG was performed according to clinical needs. All ECG and UCG records were sent to Fuwai Hospital and re-interpreted by specialists at the Department of Cardiology. The following parameters were analyzed: heart rate (HR), PR interval, QRS duration, and QT(c) interval. We classified cardiac events by ST-T segment abnormalities, elevated myocardial enzymes, arrhythmia, and QRS pattern or duration abnormalities. To clarify the relationship between treatment duration and ECG abnormalities, we defined early abnormalities as those appearing before four cycles of chemotherapy.

### Candidate SNP selection and genotyping

In total, 25 SNPs related to autophagy were selected according to the National Center for Biotechnology Information (NCBI) SNP database (https://www.ncbi.nlm.nih.gov/snp/) and the Catalog of Somatic Mutations in Cancer (COSMIC) database (http://cancer.sanger.ac.uk/cosmic). The final candidate SNPs were ataxia telangiectasia mutated (*ATM*) (rs1003623, rs227060, rs228589, rs664143, and rs664677), autophagy-related (*ATG*)*5* (rs473543 and rs3761796), *ATG7* (rs2594971, rs111595248, and rs4684789), *ATG12* (rs1058600 and rs5870670), *ATG13* (rs13448 and rs10838611), microtubule-associated protein 1 light chain (*MAP1LC*)-*3A* (rs4911429 and rs6088521), *MAP1LC*-*3B* (rs9903, rs35227715, rs7865, and rs16944733), caspase 3 (*CASP3*) (rs1049216, rs12108497, and rs2720376), crystallin alpha B (*CRYAB*) (rs14133), and stathmin 1 (*STMN1*) (rs182455).

Genomic DNA was extracted from a 1- to 2-mL blood sample that was collected from each patient upon recruitment using a blood DNA kit (BioTeKe Corporation, Beijing, China). A MassARRAY MALDI-TOF System (Sequenom Inc., San Diego, CA, USA) was used to genotype candidate SNPs according to the protocol. The PCR primers and probes (forward 5′-ACGTTGGATGAGTTTCCTCGCTCCTGTTTC-3′ and reverse 5′-ACGTTGGATGCTCTCTCTCTGGATCTGCTC for *ATG13* rs10838611; see other probes in Additional file 1: Table S1) were designed according to Assay design 3.1 (Sequenom Inc.) and synthesized by the Beijing Genomics Institute (Beijing, China).

Purified primer extension reaction products were dispensed onto a 384-well Spectro CHIP bioarray using a MassARRAY Nanodispenser RS1000 (Sequenom Inc.) and determined using a matrix-assisted laser desorption/ionization time-off light mass spectrometer. Genotype analysis was performed through the MassARRAY Typer software version 4.0 (Sequenom Inc.). Duplicate samples and negative controls (without DNA) were used for quality control of genotyping. Concordance for duplicate samples was 100% for all assays. The group information of each sample was concealed for genotyping analysis.

### Statistical analyses

We used paired-sample *T* tests to compare the differences in HR, PR interval, QRS duration, and QT(c) interval before and after chemotherapy. Chi squared test (Pearson’s *χ*^2^ test) or binary logistic regression was employed to determine the relationship among abnormalities on ECG or UCG records, demographic characteristics, and drugs used. Continuous correction of the Chi squared test was used when necessary. The Hardy–Weinberg equilibrium test was performed to validate the genotype distributions of each SNP using the *χ*^2^ test. The association between abnormalities on ECG or UCG records and genotype distributions of SNPs was estimated by calculating odds ratios (ORs) and their 95% confidence intervals (95% CIs) with multivariate logistic regression analysis. A P value of less than 0.05 was considered statistically significant, and all statistical tests were two-sided. All analyses were performed with SPSS 19.0 (IBM Inc., Chicago, IL, USA) software.

## Results

### Patient characteristics

**Fig. 1:**
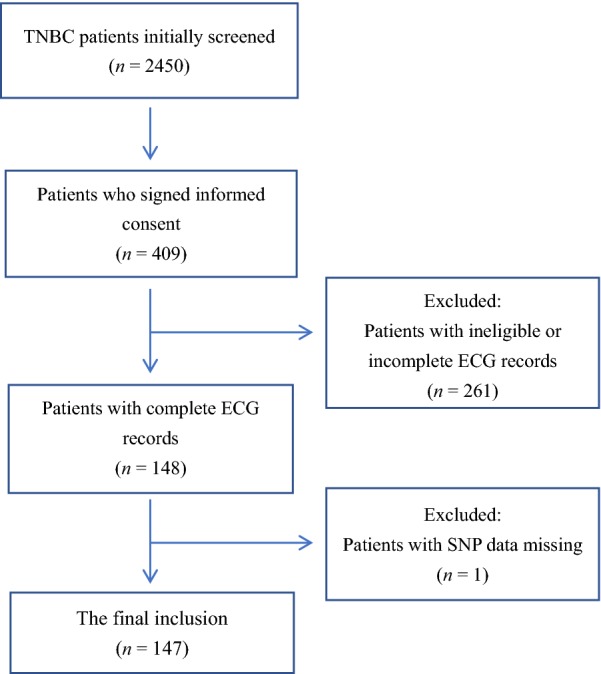
Flowchart of TNBC patient enrollment for the SNP test. *TNBC* triple-negative breast cancer, *ECG* electrocardiography, *SNPs* single nucleotide polymorphisms

From a total of 2450 stage I–III TNBC patients, 409 signed consent forms to participate in the study. Furthermore, 147 TNBC patients had relatively complete ECG records and were finally enrolled (Fig. 1). All patients were Han Chinese. The median age of the patients was 51 (range 30–75) years. The basic characteristics of patients with or without electrocardiographic changes are presented in Table 1. Among the 147 TNBC patients, only 46 (31.3%) had normal ECG records after each chemotherapy cycle. For the 16 patients who underwent UCG, 2 (12.5%) had a reversible decrease of more than 10% in LVEF with no cardiovascular symptoms. The rate of anthracycline administration was significantly lower in patients with normal ECG than in those with abnormal ECG records (47.8% vs. 66.3%, *P* = 0.033). The rate of excessive alcohol consumption (corresponding to more than 10 g of ethanol per day for females [15]) in the normal ECG group was also significantly lower than that in the abnormal ECG group (2.2% vs. 15.8%, *P* = 0.034). The differences in hypertension and diabetes, potential risk factors for abnormal ECG, were not statistically significant because of the relatively small number of patients.

**Table 1:** Clinicopathological characteristics of 147 triple-negative breast cancer (TNBC) patients with normal or abnormal electrocardiography (ECG)

Characteristic	Normal ECG group	Abnormal ECG group	*P* value
Total [cases (%)]	46 (31.3)	101 (68.7)	–
Age [years, median (range)]	51 (30–75)	50 (32–72)	0.731
Baseline heart rate [bpm, mean ± SD]	75.1 ± 10.7	75.3 ± 11.7	0.931
Disease-free survival [months, median (range)]	64.3 (7.7–183.2)	58.6 (3.6–181.7)	0.473
Hypertension [cases (%)]	5 (10.9)	17 (16.8)	0.490
Diabetes [cases (%)]	1 (2.2)	7 (6.9)	0.250
Smoking [cases (%)]	2 (4.3)	5 (5.0)	0.874
Excessive alcohol consumption [cases (%)]	1 (2.2)	16 (15.8)	0.034
Family history of heart disease [cases (%)]	4 (8.7)	7 (6.9)	0.969
Chemotherapy [cases (%)]			1.000
Neoadjuvant	3 (6.5)	5 (5.0)	
Adjuvant	43 (93.5)	96 (95.0)	
Drug uses [cases (%)]			
Anthracyclines	22 (47.8)	67 (66.3)	0.033
Cyclophosphamide	24 (52.2)	57 (56.4)	0.630
Paclitaxel	20 (43.5)	42 (41.6)	0.829
Docetaxel	19 (41.3)	40 (39.6)	0.845
Carboplatin	16 (34.8)	27 (26.7)	0.320
5-Fluorouracil	1 (2.2)	4 (4.0)	0.949
Chemotherapy regimens [cases (%)]			0.242
Anthracycline-based	6 (13.0)	16 (15.8)	
Taxane-based	23 (50.0)	33 (32.7)	
Anthracycline–taxane-based	16 (34.8)	50 (49.5)	
Others	1 (2.2)	2 (2.0)	

*SD* standard deviation

### Parametric analysis of ECG records

**Fig. 2:**
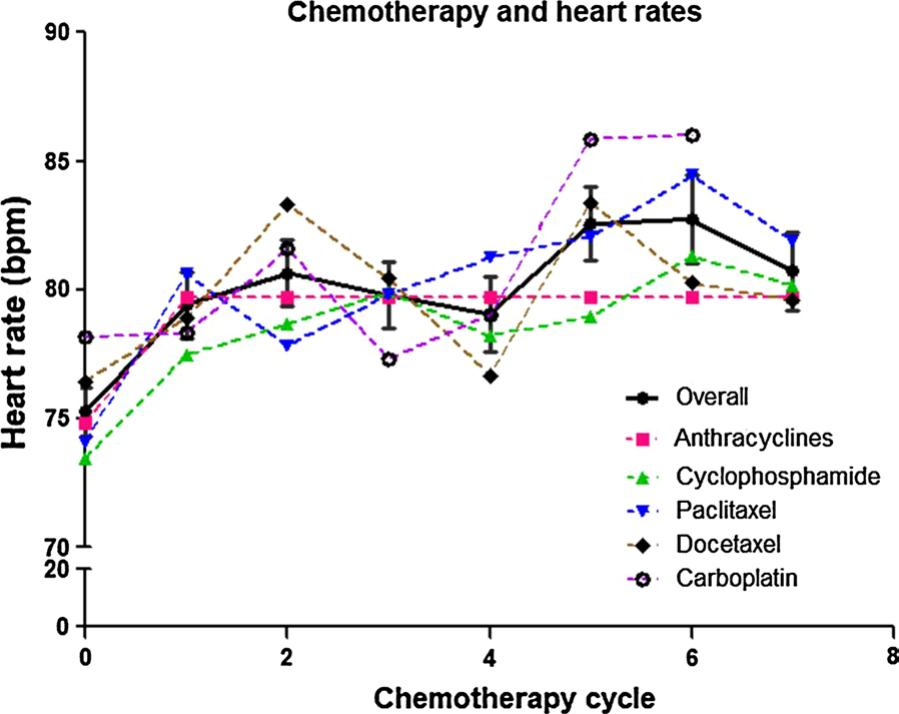
The association between heart rate changes in TNBC patients and chemotherapy drugs. No matter what the chemotherapy drugs are, the heart rate of the patients is increased with nearly every chemotherapy cycle compared with baseline. *TNBC* triple-negative breast cancer

For the 147 TNBC patients, we collected 686 ECG records. We did not include the data after the eighth cycle of chemotherapy in our analysis because of limited data and different ECG recording times. As shown in Fig. 2 and Table 2, the heart rate of patients not only increased with almost every chemotherapy cycle compared with baseline but also showed a gradual increasing trend with the continuation of chemotherapy. In patients who were treated with anthracyclines or cyclophosphamide, the heart rate was significantly increased after every cycle, whereas the increases were not significant in patients who were treated with paclitaxel, docetaxel, or carboplatin. However, no meaningful positive association was found regarding PR interval, QRS duration, and QT(c) interval (data not shown).

**Table 2:** Heart rates of TNBC patients after cycles of chemotherapy

Regimen	Time point							
	Baseline	Cycle 1	Cycle 2	Cycle 3	Cycle 4	Cycle 5	Cycle 6	Cycle 7
Overall								
Cases (%)	142 (96.6)	85 (57.8)	90 (61.2)	87 (59.2)	85 (57.8)	73 (49.7)	60 (40.8)	34 (23.1)
Heart rate (bpm, mean ± SD)	75.3 ± 11.4	79.4 ± 11.8	80.6 ± 12.3	79.8 ± 12.2	79.0 ± 13.4	82.6 ± 12.2	82.7 ± 13.3	80.7 ± 12.0
*P* value	Ref	< 0.001	< 0.001	< 0.001	< 0.001	< 0.001	< 0.001	0.003
Anthracyclines								
Cases (%)	87 (97.8)	57 (64.0)	58 (65.2)	56 (62.9)	54 (60.7)	45 (50.6)	41 (46.1)	33 (37.1)
Heart rate (bpm, mean ± SD)	74.8 ± 11.1	79.7 ± 11.7	80.1 ± 11.5	80.5 ± 11.8	79.7 ± 14.9	81.4 ± 11.3	82.7 ± 12.2	80.7 ± 12.1
*P* value	Ref	0.001	< 0.001	< 0.001	0.008	< 0.001	< 0.001	0.004
Cyclophosphamide								
Cases (%)	80 (98.8)	50 (61.7)	49 (60.5)	49 (60.5)	44 (54.3)	36 (44.4)	39 (48.1)	30 (37.0)
Heart rate (bpm, mean ± SD)	73.5 ± 11.2	77.4 ± 12.0	78.7 ± 12.7	79.9 ± 13.1	78.2 ± 10.1	78.9 ± 11.8	81.3 ± 13.5	80.2 ± 12.1
*P* value	Ref	0.004	< 0.001	< 0.001	< 0.001	< 0.001	< 0.001	0.001
Paclitaxel								
Cases (%)	60 (96.8)	37 (59.7)	39 (62.9)	39 (62.9)	43 (69.4)	28 (45.2)	28 (45.2)	18 (29.0)
Heart rate (bpm, mean ± SD)	74.1 ± 10.0	80.6 ± 12.1	77.8 ± 10.9	79.8 ± 10.5	81.2 ± 9.0	82.0 ± 10.5	84.4 ± 14.2	81.9 ± 12.7
*P* value	Ref	< 0.001	< 0.001	0.002	< 0.001	< 0.001	0.002	0.086
Docetaxel								
Cases (%)	56 (94.9)	35 (59.3)	39 (66.1)	36 (61.0)	34 (57.6)	36 (61.0)	24 (40.7)	14 (23.7)
Heart rate (bpm, mean ± SD)	76.4 ± 13.2	78.9 ± 12.3	83.3 ± 14.0	80.4 ± 14.9	79.0 ± 12.2	83.4 ± 13.9	80.3 ± 13.5	79.6 ± 12.2
*P* value	Ref	0.137	0.001	0.037	0.036	0.004	0.231	0.004
Carboplatin								
Cases (%)	40 (93.0)	20 (46.51)	25 (58.1)	23 (53.5)	25 (58.1)	26 (60.5)	14 (32.6)	NA
Heart rate (bpm, mean ± SD)	76.1 ± 11.6	78.3 ± 9.2	81.6 ± 11.3	77.3 ± 9.9	79.0 ± 9.1	85.8 ± 11.9	86.0 ± 14.5	NA
*P* value	Ref	0.442	0.023	0.690	0.034	0.001	0.157	NA

All data are compared with baseline values. NA, not applicable (platinum-based chemotherapy was used for only 4–6 cycles)

### Cardiac events

Among the 101 TNBC patients with abnormal ECGs, ST-T segment abnormalities were observed on 45 (44.6%) patients, elevated myocardial enzymes on 4 (4.0%), arrhythmia on 52 (51.5%), and QRS pattern or duration abnormalities on 14 (13.9%). ST-T segment abnormalities included ST segment depression and elevation, as well as flat, inverted, and bidirectional T waves. Fourteen (13.9%) patients had two types of cardiac event at the same time. However, among the 45 patients with ST segment abnormalities, only 4 patients displayed clinically significant changes in ST segments, and their electrocardiograms turned normal after postponing chemotherapy and adding secondary prevention drugs for coronary heart disease. Among the 52 patients with arrhythmia, sinus tachycardia was the most common (37, 71.2%), followed by sinus irregularity (8, 15.4%), sinus bradycardia (4, 7.7%), premature ventricular contraction (3, 5.8%), premature atrial contraction (2, 3.8%), complete right bundle branch block (2, 3.8%), atrial flutter and junctional premature beating (1, 1.9%). Among the 16 patients who underwent UCG, 2 (12.5%) had a significant reversible decrease of more than 10% in LVEF. The LVEF of the 2 patients became greater than 45% after treatment for heart failure, and the subsequent treatment was administered as scheduled.

The use of anthracyclines was associated with QRS duration abnormalities (*P *< 0.05), whereas the use of CBP was associated with reduced frequencies of ST-T segment abnormalities (*P *< 0.05) (Table 3). Anthracycline-based regimens were associated with ST-T segment abnormalities (*P *< 0.05), whereas taxane-based regimens were associated with QRS duration abnormalities (*P *< 0.05) (Table 4). However, in an effort to explore the timing of cardiac events, there was no difference in cardiac events during or after the first four cycles.

**Table 3:** Relationships between ECG abnormalities and chemotherapeutic drugs

Drug	Total (cases)	ST-T segment abnormalities			Elevated myocardial enzymes			Arrhythmia			QRS pattern or duration abnormalities		
		No. of events [cases (%)]	χ^2^	*P* value	No. of events [cases (%)]	χ^2^	*P* value	No. of events [cases (%)]	χ^2^	*P* value	No. of events [cases (%)]	χ^2^	*P* value
Anthracyclines	89	32 (36.0)	3.031	0.082	3 (3.4)	0.007	0.935	30 (33.7)	0.274	0.601	12 (13.5)	4.104	*0.043*
Cyclophosphamide	81	27 (33.3)	0.629	0.428	2 (2.5)	0.000	1.000	28 (34.6)	0.051	0.821	10 (12.3)	1.667	0.197
Paclitaxel	62	18 (29.0)	0.126	0.723	2 (3.2)	0.000	1.000	20 (32.3)	0.455	0.500	6 (9.7)	0.003	0.957
Docetaxel	59	13 (22.0)	3.414	0.065	2 (3.4)	0.000	1.000	25 (42.4)	2.112	0.146	4 (6.8)	0.411	0.521
Carboplatin	43	8 (18.6)	4.126	*0.042*	2 (2.3)	0.135	0.713	16 (37.2)	0.090	0.765	2 (4.6)	0.971	0.324

**Table 4:** Relationships between ECG abnormalities and chemotherapy regimens

Chemotherapy regimen	Total (cases)	ST-T segment abnormalities			Elevated myocardial enzymes			Arrhythmia			QRS pattern or duration abnormalities		
		No. of events [cases (%)]	χ^2^	*P* value	No. of events [cases (%)]	χ^2^	*P* value	No. of events [cases (%)]	χ^2^	*P* value	No. of events [cases (%)]	χ^2^	*P* value
Anthracycline-based	22	12 (54.5)	6.977	*0.008*	0 (0)	0.020	0.889	4 (18.2)	2.519	0.112	4 (18.2)	1.224	0.269
Taxane-based	56	13 (23.2)	2.331	0.127	1 (1.8)	0.001	0.980	21 (37.5)	0.179	0.672	2 (3.6)	2.687	*0.001*
Anthracycline–taxane-based	66	19 (28.8)	0.188	0.665	3 (4.5)	0.515	0.430	25 (37.9)	0.329	0.566	8 (12.1)	0.938	0.333
Others	3	1 (33.3)	0.000	1.000	0	0.000	1.000	2 (66.7)	0.287	0.592	0	0.000	1.000

### Autophagy-related SNPs and ECG abnormalities

All selected patients underwent SNP test. According to previous studies, we selected 25 SNPs associated with autophagy.

**Fig. 3:**
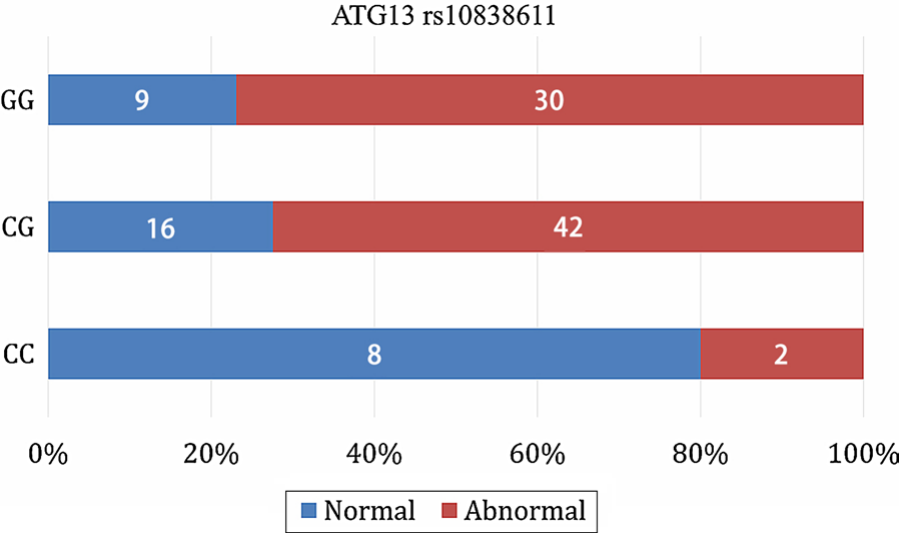
Association between *ATG13* rs10838611 genotype and ECG abnormalities in TNBC patients. Patients with GG genotype of *ATG13* rs10838611 had significantly higher rate of ECG abnormalities than did those with CC or CG genotype (odds ratio [OR] = 2.258, 95% confidence interval (CI) = 1.318–3.869; *P *= 0.003)

The proportion of patients with ECG abnormalities was different among the three *ATG13* rs10838611 genotype groups (Fig. 3). The G allele of *ATG13* rs10838611 was significantly associated with ECG abnormalities (OR = 2.258, 95% CI = 1.318–3.869; *P *< 0.01), indicating an increased risk of cardiac events. However, the other SNPs did not display significant associations with ECG abnormalities (Table 5).

**Table 5:** Associations of 25 SNPs of 10 genes with ECG abnormalities

Gene	SNP	Nucleotide change	MAF	HWE *P* value	*χ* ^2^	Association *P* value
ATM	rs1003623	C/T	0.49	0.00	1.57	0.46
	rs227060	C/T	0.32	0.50	0.85	0.65
	rs228589	A/T	0.46	0.00	0.82	0.57
	rs664143	C/T	0.37	0.93	0.20	0.91
	rs664677	A/C/T	0.35	0.23	0.95	0.62
ATG5	rs473543	C/T	0.39	0.51	1.42	0.49
	rs3761796	A/G	0.07	0.25	2.06	0.36
ATG7	rs2594971	C/T	0.50	0.01	0.37	0.83
	rs111595248	C/T	0.05	0.48	0.33	0.56
	rs4684789	G/T	0.42	0.34	0.50	0.78
ATG12	rs1058600	A/G	0.42	0.96	1.77	0.41
	rs5870670	Del/T	0.39	–	1.37	0.24
STMN1	rs182455	C/T	0.36	0.35	2.84	0.24
ATG13	rs13448	C/T	0.40	0.20	3.25	0.20
	rs10838611	C/G/T	0.38	0.08	20.19	*0.00*
MAP1LC3A	rs4911429	A/G	0.36	0.75	1.17	0.56
	rs6088521	A/C/G/T	0.35	0.43	0.67	0.72
MAP1LC3B	rs9903	C/T	0.19	0.38	0.55	0.76
	rs35227715	C/G	0.25	0.12	0.53	0.77
	rs7865	C/G/T	0.31	0.00	0.57	0.45
	rs16944733	A/C/T	0.20	0.00	1.58	0.45
CASP3	rs1049216	C/T	0.40	0.58	1.75	0.42
	rs12108497	C/G/T	0.39	0.25	0.97	0.61
	rs2720376	C/T	0.46	0.25	0.46	0.79
CRYAB	rs14133	C/G	0.24	0.05	6.31	0.10

*HWE* Hardy–Weinberg equilibrium, *MAF* minor allele frequency, *ATM* ataxia telangiectasia mutated, *ATG* autophagy-related, *MAP1LC* microtubule-associated protein 1 light chain, *CASP* caspase, *CRYAB* crystallin alpha B, *STMN1* stathmin 1

Because of the differences in the mechanisms of cardiotoxicity of various chemotherapy drugs, we performed subgroup analysis to determine their relationships with autophagy-related SNPs. With simple data analysis, SNP genotype differences in *MAP1LC3B* (rs7865 and rs9903) in the anthracyclines subgroup, *ATM* (rs1003623) and *ATG13* (rs10838611) in the CTX subgroup, *MAP1LC3B* (rs7865) in the PTX subgroup, and *MAP1LC3A* (rs6088521) in the DTX subgroup demonstrated potential associations with cardiac toxicity. However, after Hardy–Weinberg equilibrium test and detailed genotypic analysis of each SNP, we considered that these associations were likely false positives. Therefore, no associations between SNPs and chemotherapy drugs were identified.

## Discussion

In the present study, we demonstrated that ECG abnormalities were caused by chemotherapy, often asymptomatic, more common than expected. Only 46 (31.3%) patients had normal electrocardiograms after each chemotherapy cycle, consistent with the rate observed by Carver et al. [16]. These data indicate that chemotherapy-induced cardiac adverse events are common and need to be monitored in the real world.

We also found that patient heart rate increased during chemotherapy. High resting heart rate is a known cardiovascular disease risk factor [17] and increases the relative risk of all-cause mortality [18]. The elevated heart rate is another factor illustrating the adverse effects of chemotherapy drugs on the cardiovascular system. Recent studies have demonstrated that a high heart rate would increase the incidence and mortality of cancer [19, 20]. However, the measurement of heart rate is influenced by many factors, and increased heart rate could also be a marker of stress, physical activity, cigarette smoking, blood pressure level, blood viscosity, and plasma level of catecholamines [19, 20]; thus, its clinical predictive value is limited.

All SNPs investigated in the present study have been confirmed to be associated with autophagy. Autophagy has dual functions. Under physiological conditions, autophagy is essential for maintaining normal functions of cells and for removing excess proteins. Under pathological conditions, autophagy can be abnormally activated by stressors of survival and can eventually lead to cell death [10]. Dysregulation of autophagy is one of numerous mechanisms of cardiotoxicity. The present study confirmed the relationship between autophagy-related SNPs and ECG abnormalities and provided new evidence connecting autophagy to cardiac damage caused by chemotherapy.

The only identified SNP associated with ECG abnormalities is located in the *ATG13* gene. ATG proteins regulate autophagosome formation. Among the 35 ATG proteins identified in yeast thus far, ATG1–10, 12–14, 16, and 18 comprise the core machinery for membrane formation [11, 21]. In addition to being a core member, ATG13 functions as a regulator of other ATG family members, such as ATG1, and thus plays an important role in autophagy [21]. However, at present, there are no studies on the relationship between chemotherapy-induced cardiac events and the ATG family. The present study demonstrated the association between cardiac adverse events and autophagy-related SNPs, providing not only evidence for the involvement of autophagy in the cardiotoxicity of chemotherapy but also ideas for subsequent research.

In previous studies, many SNPs, such as retinoic acid receptor gamma (*RARG*) rs2229774 [22], UDP glucuronosyltransferase family 1 member A6 (*UGT1A6*) rs6759892, spastic paraplegia 7 (*SPG7*) rs2019604 [14, 23], and solute carrier family 28 member 3 (*SLC28A3*) rs7853758 [23], have been associated with cardiotoxicity. As increasing SNPs related to the cardiotoxicity of chemotherapy are discovered, along with the novel SNP found in the present study, we may be able to establish a model to predict the susceptibility of cancer patients to cardiac events and help guide clinical treatment.

Hardy–Weinberg equilibrium (HWE), also known as the law of genetic equilibrium, is the most important principle in population genetics. In the present study, we found that the HWE *P* values of several SNP loci were < 0.05. One possible cause may be that the study participants were breast cancer patients rather than healthy individuals. In addition, the relatively small sample size may also lead to this phenomenon.

Nevertheless, several limitations existed in the present study. First, there might be an inherent selection bias because all patients were from single hospital. However, at the National Cancer Center, patients come from all over China, which may minimize this bias. Second, UCG was performed according to clinical needs rather than a regular requirement. However, most of the recruited patients were at early stages, and the cumulative dose of chemotherapy drugs was low, which may have resulted in a lack of significant cardiotoxicity and thus a low number of UCG records collected [7, 8]. Third, the ECG abnormalities in the present study were mainly non-specific, making the clinical significance of the results less relevant. Last, the relative small sample size might have limited the statistical power of the present study.

## Conclusions

Our study not only investigated the occurrence of early cardiac events caused by chemotherapy in TNBC patients but also identified some differences in clinical characteristics and risk factors associated with ECG abnormalities. Upon autophagy-related SNP detection, we found carriers of the G allele of *ATG13* rs10838611 to have higher frequencies of cardiac events. Our findings may help clinicians better screen high-risk groups for chemotherapy-induced cardiac events and improve treatment decision for cancer patients.

## Additional file

**Additional file 1: Table S1.** The polymerase chain reaction primers and probes involved.

## Abbreviations

TNBC: triple-negative breast cancer
ECG: electrocardiography
UCG: echocardiography
SNP: single nucleotide polymorphisms
IHC: immunohistochemistry
FISH: fluorescence in situ hybridization
EPI: epirubicin
PTX: paclitaxel
DTX: docetaxel
CTX: cyclophosphamide
CBP: carboplatin
5-FU: 5-fluorouracil
HR: heart rate
LVEF: left ventricular ejection fraction
